# Neurocognitive impairment in childhood chronic fatigue syndrome

**DOI:** 10.3389/fphys.2013.00087

**Published:** 2013-04-19

**Authors:** Kei Mizuno, Yasuyoshi Watanabe

**Affiliations:** ^1^Pathophysiological and Health Science Team, RIKEN Center for Life Science TechnologiesKobe City, Hyogo, Japan; ^2^Department of Physiology, Osaka City University Graduate School of MedicineOsaka, Japan; ^3^Department of Medical Science on Fatigue, Osaka City University Graduate School of MedicineOsaka, Japan

**Keywords:** childhood chronic fatigue syndrome, cognitive development, divided attention, event-related potential, functional magnetic resonance imaging, kana pick-out test, modified advanced trail making test, switching attention

## Abstract

Neurocognitive impairment is a feature of childhood chronic fatigue syndrome (CCFS). Several studies have demonstrated reduced attention control in CCFS patients in switching and divided attention tasks. In students, the extent of deterioration in task performance depends on the level of fatigue. Poor performance in switching and divided attention is common in both fatigued students and CCFS patients. Additionally, attentional functions show dramatic development from childhood to adolescence, suggesting that abnormal development of switching and divided attention may be induced by chronic fatigue. The brain structures associated with attentional control are situated in the frontal and parietal cortices, which are the last to mature, suggesting that severe fatigue in CCFS patients and students may inhibit normal structural and functional development in these regions. A combination of treatment with cognitive behavioral therapy and antidepressant medication is effective to improve attentional control processing in CCFS patients. Studies identifying the features of neurocognitive impairment in CCFS have improved our current understanding of the neurophysiological mechanisms of CCFS.

## Introduction

Myalgic encephalopathy (ME), also known as chronic fatigue syndrome (CFS), is a critical and relatively common condition in children and adolescents as well as in adults. ME/CFS is the largest single cause of long-term school absence in the UK (Dowsett and Colby, 1997), and affects between 0.1 and 2% of children under 18 years of age in the US. (Chalder et al., 2003; Rimes et al., 2007), and 2.6% of junior high and 5–10% of senior high school students suffer from childhood CFS (CCFS) in Japan (Miike et al., 2004).

For a diagnosis of CFS, most researchers use criteria that were developed by the International CFS Study Group (Fukuda et al., 1994) that was established for adults rather than children. In 2006, diagnostic criteria for children and adolescents with ME/CFS were developed by the International Association of CFS Pediatric Case Definition Working Group (Jason et al., 2006). The symptom categories included fatigue, post-exertional malaise, unrefreshing sleep or disturbance of sleep quantity, pain (myofascial, joint, abdominal, and/or head pain), two or more neurocognitive manifestations (impaired memory, difficulty focusing, difficulty finding the right word, absentmindedness, slowness of thought, difficulty recalling information, difficulty focusing on one thing at a time, trouble expressing thought, difficulty comprehending information, frequently losing train of thought, and/or new trouble with math or other educational subjects).

Among the cognitive impairments associated with CCFS, memory and concentration problems affect over 80% of patients (Miike et al., 2004). However, it is difficult to identify these impairments of CCFS using cognitive tests (Kawatani et al., 2011). Several studies have revealed that performance is reduced in attentional control tasks in CCFS patients (Tomoda et al., 2007; Haig-Ferguson et al., 2009; Kawatani et al., 2011). In this review, we discuss the neural mechanisms of cognitive impairments in CCFS from the standpoint of performance on cognitive tasks and neurophysiological responsiveness such as event-related potentials (ERP) of electroencephalogram and activations and neural circuits of brain regions using functional magnetic resonance imaging (fMRI). In addition, we discuss the effects of treatment on decreased cognitive function in CCFS patients.

## Attentional control impairment

The number of reports related to cognitive dysfunction associated with CCFS using cognitive tests is limited (Haig-Ferguson et al., 2009; Kawatani et al., 2011). Haig-Ferguson et al. (2009) used a battery of 10 tests to measure processing speed, attention, immediate and delayed memory, working memory, and executive function in CCFS patients. They found that CCFS patients have problems with attention control, such as sustained attention, switching attention, divided attention, and auditory learning. We used a battery of 6 tests to measure motor skill, selective, switching and divided attention, and working memory of patients with CCFS (Kawatani et al., 2011). We demonstrated that performance is reduced in CCFS patients on switching attention using a modified Advanced Trail Making Test (mATMT), which is a visual search task (Kajimoto, 2008; Mizuno and Watanabe, 2008; Kawatani et al., 2011). Previously, we demonstrated that performance is impaired on a divided attention task, using the kana-pick out test (KPT), which is a dual verbal task (Kaneko, 1996; Tomoda et al., 2007). These studies indicate that attentional control is reduced in CCFS, including switching and dividing attention.

When students proceed to junior high school from elementary school, they encounter rapid environmental changes, which can cause a variety of behavioral and emotional problems (Spear, 2000). One example is the number of Japanese students with school-refusal, which is reported to be 7154 out of 1,201,134 (0.5%) in 6th-graders and 21,084 out of 1,177,557 (1.8%) in 7th-graders in 2010 (MEXT, 2010). Thus, the prevalence of CCFS included in school-refusal increases from elementary school to junior high school, indicating that the number of fatigued students increases from elementary school to junior high school (Mizuno et al., 2011a). Recent results demonstrated that performance on switching and divided attention on the mATMT and KPT, respectively, in fatigued students in junior high school was reduced relative to controls, similar to performance in CCFS patients (Mizuno et al., 2011a). Thus, decreased performance on switching and divided attention tasks appears to be a common characteristic of both fatigued students and CCFS patients.

## Development of attentional control

Morphological analyses in children and adolescents have shown that brain maturation occurs at different rates in different brain regions. The primary sensory and motor areas are the first to mature, while the association areas, especially in the frontal and parietal regions, are the last to mature (Sowell et al., 1999; Gaillard et al., 2001). Along with structural changes in the brain, executive function (defined as the set of mental control processes that permit goal-directed behavior) develops dramatically between childhood and adolescence (Travis and Tecce, 1998). In the development of executive function, for example, age-related gain has been reported in inhibitory control (Van der Molen, 2000), working memory (Casey et al., 2000; Vuontela et al., 2003), task switching (Cepeda et al., 2000), adaptive problem solving (Chelune and Baer, 1986), and various other planning and problem solving tasks (Welsh et al., 1991). Executive function is also related to the control of attention (Cowan et al., 2005), an important element of information processing that is embodied in the central executive component in theoretical conceptions of working memory (Cowan, 1988). Attentional competency develops steadily through early and late childhood, perhaps due in part to the development of core processing resources (Bisanz et al., 1979).

For switching and divided attention, we demonstrated that performance on motor processing tasks of students improved by grade in elementary school. However, performance did not change from elementary school to junior high school (Mizuno et al., 2011b). In contrast, performance of students on working memory, switching and divided attention tasks improved from elementary to junior high school (Mizuno et al., 2011b). In junior high school, performance on divided attention tasks improved by grade (Mizuno et al., 2011b). Taken together, these results suggest that fatigue influences the age-dependent development of attention control in students and CCFS patients.

## Brain mapping for attention control

Neuroimaging studies using fMRI have revealed the neural structures associated with switching attention (Zakzanis et al., 2005) and divided attention (Mizuno et al., 2012a). Task E in the mATMT was reported to measure the ability to switch attention while performing task B of the trail making test (Reitan, 1955). It was observed to be comparable to task E, activation in the left prefrontal cortex, including the dorsolateral prefrontal cortex, and ventrolateral prefrontal cortex (Zakzanis et al., 2005). Processing of divided attention in the KPT requires activation of the left frontal and parietal cortices, including the dorsal inferior frontal gyrus and superior parietal lobule, in young adults (Mizuno et al., 2012a). In addition, we observed increased synchronization between these brain regions during the KPT, suggesting that effective communication between them may be induced, which may contribute to more attentional processing (Mizuno et al., 2012a). Clinical studies have validated the use of the most extensively studied cognitive ERP component, the P300, as an indicator of perceptual, attentional, and short-term memory processes on an oddball task (Sutton et al., 1965). In CCFS patients, we found that prolonged P300 latency during a visual oddball task is related to a decrease in performance on the KPT (Tomoda et al., 2007). These results suggest that there is a delay of neural processing and/or lower synchronization between brain regions that are associated with decreased performance during divided attention. Since the frontal and parietal cortices are the last to mature from childhood to adulthood (Sowell et al., 1999; Gaillard et al., 2001), it is possible that severe fatigue in students and CCFS patients inhibits structural and functional development in these regions. In adults with CFS, a decrease in divided attention (Ross et al., 2001) and a reduction of gray matter volumes in the lateral prefrontal cortex have been observed (Okada et al., 2004; de Lange et al., 2008), which support this possibility.

## Treatment effects on attention control

**Figure 1:**
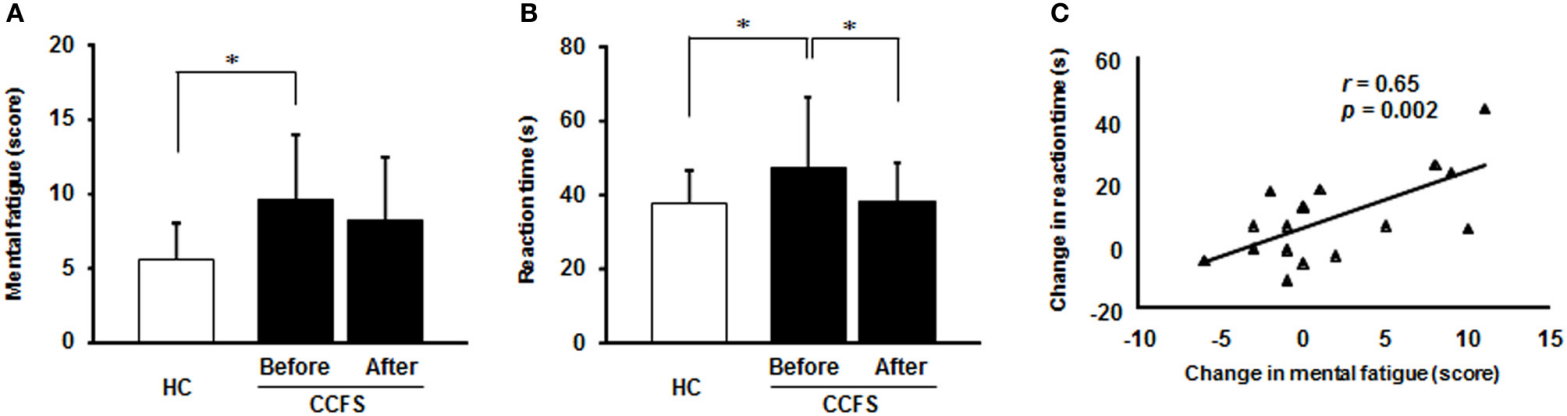
Effects of combination treatments of cognitive behavioral therapy and antidepressant medication (fluvoxamine) for 6 months on (A) the severity of mental fatigue and (B) task performance on a attention switching task in patients with childhood chronic fatigue syndrome (CCFS). **(C)** Correlation between changes in mental fatigue score and reaction time from before to after the treatment in CCFS patients. HC, healthy control. Values are means and error bars indicate SD. Statistically significant, ^*^p < 0.05.

No clearly effective therapy is available for CCFS patients (Miike et al., 2004), but data from a brain morphometry study has suggested that cognitive behavioral therapy (CBT), which combines a rehabilitative approach of a graded increase in physical activity with a psychological approach, may be beneficial (de Lange et al., 2008). However, CBT has been described as ineffective, not evidence-based, and potentially harmful for CFS treatment (Twisk and Maes, 2009). Many pharmacological therapies have been used for treating CFS including anti-depressants, non-steroidal anti-inflammatory drugs, anxiolytic drugs, anti-allergy drugs, anti-hypotensive drugs, and antidepressants such as selective serotonin reuptake inhibitors (SSRIs). However, such medications are not universally beneficial (Afari and Buchwald, 2003). A recent randomized controlled study in youths with depression who had not improved during a SSRI trial showed that cognitive performance can be moderated by combined treatment with CBT and antidepressant medication, suggesting that CBT combined with medication may be more advantageous for depressive adolescents than medication or CBT alone (Asarnow et al., 2009). Recently, we investigated the effects of combination therapy with CBT and antidepressant medication on the severity of mental fatigue, which was evaluated by Chalder's fatigue scale (Chalder et al., 1993; Tanaka et al., 2008), and cognitive performance in CCFS patients (Kawatani et al., 2011). This combination therapy decreased the severity of mental fatigue (Figure 1A) and improved performance on switching attention tasks and on the mATMT (Figure 1B). The association between these changes in fatigue severity and task performance by treatment are shown in Figure 1C. These results suggest that combined treatment with CBT and medication is effective for improving poor attention characteristics associated with CCFS.

## Summary and future study

Several studies have shown that CCFS patients have poor performance on attention control tasks such as attention switching, and divided attention on the mATMT and KPT, respectively. In students, performances on switching and divided attention tasks are also reduced, the extent of which is dependent on the fatigue level. Therefore, deterioration on switching and divided attention tasks are a common characteristic feature in both fatigued students and CCFS patients. Additionally, these attentional functions are dramatically developed from childhood to adolescence, suggesting that inappropriate development of switching and divided attention is induced by fatigue. The neural structures associated with attention switching and divided attention are regions of the frontal and parietal cortices, which are the last to mature from childhood to adulthood, suggesting that severe fatigue of CCFS patients and students may inhibit structural and functional development in these regions. For CCFS patients, combined treatment with CBT and antidepressant medication is effective to improve attentional control processing.

In addition to neurocognitive impairments associated with CCFS, impairments of autonomic function include decreased parasympathetic activity and increased relative sympathetic activity (Tomoda et al., 2007). These symptoms also occur in adults with CFS (Wyller et al., 2007; Burton et al., 2010). Sympathoexcitatory subcortical threat circuits are normally under the inhibitory control of the prefrontal cortex (Amat et al., 2005; Thayer, 2006; Thayer and Sternberg, 2006). Previous studies showed the decreased regional cerebral blood flow of the frontal, temporal, and occipital cortices in CCFS patients using N-isopropyl-p-[^123^I]iodoamphetamine single photon emission computed tomography and xenon-computed tomography (Tomoda et al., 1995, 2000). A neurochemical investigation of the choline concentration of the frontal white matter in patients with CCFS demonstrated an increase, as measured by magnetic resonance spectroscopy (Tomoda et al., 2000). These findings indicate abnormal cerebral blood flow, cholinergic function, and autonomic function in patients with CCFS. However, the associations among decreases in attention control and these physiological and chemical functions of the brain are still unclear, and thus, further neuroimaging study is necessary for elucidating the neural and molecular mechanisms of cognitive impairments in CCFS.

In adult CFS patients, evidence such as lowered cerebral activity in the prefrontal cortex during fatigue-inducing tasks (Caseras et al., 2008) and a bilateral reduction of gray-matter volume in the prefrontal cortices (Okada et al., 2004; de Lange et al., 2008) suggest that individuals with CFS may exhibit anatomical and functional alterations in the prefrontal cortex. In childhood and adolescence, excessive stress, such as that due to verbal and sexual abuse, is associated with alterations of the brain structure (Tomoda et al., 2009, 2011). The influence of chronic fatigue on the brains of children and adolescents including CCFS patients remains unclear. Further prospective neuroimaging studies are necessary to identify the vulnerability of the lateral prefrontal cortices and other brain regions and interactions between attention control and brain structures in CCFS.

Monitoring of cognitive function in fatigued students and CCFS patients using the mATMT and KPT is useful for the evaluation of attention control. In addition, the evaluation of attention control might aid in the development of therapeutic strategies such as the CBT and medications for treating CCFS (Kawatani et al., 2011). Our recent work found that poor performance on divided attention tasks using the KPT in junior high school students also relates to poor lifestyle choices (skipping breakfast, spending too much time watching television) and family conditions (little time spent with family and little praise from family members) (Mizuno et al., 2012b). These results suggest that further studies should investigate the effects of not only CBT and medications, but also interventions of lifestyle and family condition in CCFS patients.

### Conflict of interest statement

The authors declare that the research was conducted in the absence of any commercial or financial relationships that could be construed as a potential conflict of interest.
